# Risk factors for postoperative surgical site infections in patients with Crohn’s disease receiving definitive bowel resection

**DOI:** 10.1038/s41598-017-10603-8

**Published:** 2017-08-29

**Authors:** Song Liu, Ji Miao, Gefei Wang, Meng Wang, Xiuwen Wu, Kun Guo, Min Feng, Wenxian Guan, Jianan Ren

**Affiliations:** 1 Department of General Surgery, Nanjing Drum Tower Hospital, The Affiliated Hospital of Nanjing University Medical School, Nanjing, 210008 China; 2 Department of General Surgery, Jinling Hospital, Medical School of Nanjing University, Nanjing, 210002 China

## Abstract

Surgical site infection presents as a significant problem that limits the potential benefits of surgical interventions. This study is to investigate risk factors for postoperative SSI in patients with Crohn’s disease receiving definitive bowel resection. A case-control study including 49 patients with SSI and 105 patients without SSI was performed. Demographics, clinical characteristics, laboratory information, medical and surgical data were compared between groups. Significant elements were subsequently brought into logistic regression analysis for further identification. Patients with SSI exhibited higher tobacco usage rate (*p* = 0.03), lower preoperative hemoglobin (*p* = 0.02) and pre-albumin level (*p* = 0.02). Bowel penetration instead of stricture was more frequent in patients with SSI (*p* = 0.04). Longer duration of operation (*p* = 0.03) and higher intraoperative lactate level (*p* = 0.02) were observed in patients with SSI. Logistic analysis identified that preoperative pre-albumin (95% CI: 0.2~0.7; OR = 0.5; *p* = 0.03), duration of operation (95% CI: 2.3~9.5; OR = 3.8; *p* = 0.03) and intraoperative lactate level (95% CI: 1.7~7.2; OR = 3.4; *p* = 0.04) were associated with postoperative surgical site outcome. Our data has identified that lower preoperative pre-albumin, longer duration of operation and higher intraoperative lactate level are risk factors for SSI in patients with Crohn’s disease receiving definitive bowel resection.

## Introduction

Surgical site infection (SSI) is the most frequent type of healthcare-associated infections^1^. SSI is associated with prolonged hospital stay, long-term disability and additional financial burden, and significantly hampers the potential benefits of surgical interventions. Notably, SSI is theoretically preventable but requires particular investigation of early prognosis and intervention^2^.

Crohn’s disease (CD) is characterized by chronic transmural intestinal inflammation that could cause ulceration, stricture and perforation of bowel wall^3, 4^. Therefore, approximately 70~90% patients with CD will undergo at least one gastrointestinal operation during their course of disease^5^. Furthermore, high relapse and recurrence rates result in high probability of reoperations in these patients. It is therefore particularly important for CD patients to prevent postoperative SSI to accelerate postoperative recovery, avoid incisional adhesion and hernia, and create better incisional environment for future possible reoperations.

The new WHO recommendations for SSI prevention has been recently released^6, 7^. A bundle of perspectives has been proposed to decrease the incidence of SSI. However, validation of these recommendations in Crohn’s disease has not been performed. Very few studies are designed to specifically explore the characteristics of SSI in CD patients. It is urgent to identify significant predictive factors for postoperative SSI in patients with Crohn’s disease, especially in definitive bowel resections. In current study, we will conduct a retrospective case-control study to determine risk factors of SSI, thereby providing evidences for early predict and identification of SSI in CD patients receiving definitive bowel resection.

## Materials and Methods

### Patients and data collection

Between January 2014 and December 2016, all patients with Crohn’s disease that registered in our hospital were recruited for qualification screening. The inclusion criteria were as follows: (1) patients were diagnosed as Crohn’s disease according to their clinical manifestations, radiologic, endoscopic, and/or histopathologic evidences; (2) patients received definitive bowel resection during hospitalization primarily due to bowel stricture (obstruction) or bowel penetration (fistula) caused by Crohn’s disease. A definitive bowel resection is defined as an operation in which a resection of bowel containing Crohn’s disease has occurred with apparent removal of all diseased bowel at the time of surgery^8^. Perianal involvement could coexist with intestinal lesion; (3) patients have not underwent previous bowel operation, although percutaneous drainage for abscess, surgical treatment for perianal complications and that unrelated to Crohn’s disease such as cholecystectomy and repair of incisional hernia are permitted and would be calculated into past surgical history.

Data of demographics, clinical characteristics, medical and surgical management was retrieved from the Electronic Medical Record System. Blood sample was collected just before the definitive surgery. SSI in current study was defined in accordance to the criteria released by WHO^7^, *i.e*., an infection that occurred at the surgical site within 30 days after surgery and that was characterized by any of the following circumstance: purulent drainage from the surgical site, organism cultured from the fluid of surgical site, and/or incisional inflammation (pain, tenderness, localized swelling and redness). All three subtypes of SSI including superficial, deep and organ/space SSI were taken into account for subsequent analysis.

### Perioperative management

For all patients with Crohn’s disease in our hospital, preoperative management included induction and maintenance of remission by proper medications, preoperative nutritional support by enteral and/or parenteral nutrition, reinforcement of physical rehabilitation, as well as appropriate source control strategy such as percutaneous drainage for intra-abdominal abscess. Antibiotics would be prescribed in patients with suspicious infections such as fever, elevated WBC (white blood cells) or procalcitonin levels, and intra-abdominal infections suggested by CT scan. Definitive bowel resection would be performed when the remission of CD activity, nutritional status and effect of source control fulfilled the requirement of the surgeons.

Prophylactic antibiotics (single dose of cefuroxime) was intravenously administrated within 30 minutes before surgery^9^. Intraoperative re-administration of antibiotics would be performed in prolonged surgical procedure or significant blood loss. Broad-spectrum antibiotics was initiated when postoperative SSI occurred, and would be subsequently replaced by specific antibiotics according to microbial evidences. In addition, local dressing, suture removal, pus drainage, debridement and even open wound care would be performed according to the severity of SSI. All surgical interventions of enrolled patients were carried out by the same surgical team consisted of a gastrointestinal consultant, two full-time attendings and two residents.

### Statistics

Continuous variables were expressed as mean ± SE (standard error) and compared with unpaired student’s *t*-test in additional to Welch’s correction when equal variance was not assumed. Discrete variables were presented as frequencies (percentages), and compared with chi-square test or Fisher’s exact test. Binary logistic regression analysis with forward (conditional) stepwise selection was performed to identify significant risk factors for postoperative SSI. All variables that has been found statistically different between groups would be entered into logistic regression analysis. All statistical analysis in this study was performed within GraphPad Prism Software (version 7.0a; GraphPad, San Diego, CA, USA).

### Ethics statement

This study has been approved by the Ethics Committees of Nanjing Drum Tower Hospital and Jinling Hospital. As a retrospective study, informed content is not required from each participant.

### Data availability statement

The datasets generated during and/or analyzed during the current study are available from the corresponding author on reasonable request.

## Results

Between January 2014 and December 2016, a total of 154 patients with Crohn’s disease that received definitive bowel resection were enrolled into this study. According to the appearance of postoperative SSI, 49 patients were divided into SSI group and the other 105 patients were divided into non-SSI group. The three types of SSI (superficial, deep and organ/space) occurred in 26 (53.1%), 15 (30.6%) and 8 (16.3%) patients, respectively. All SSI were controlled or significantly improved, and no patient died from incisional infection before discharge. Male predominance and middle-age pattern were observed in both groups. Both the BMI and duration of disease were similar between two groups as well. Most of patients with Crohn’s disease (77.6% and 75.2% in each group, respectively) had past surgical history before definitive bowel resection. The incidence of SSI was similar between CD patients with and without previous surgery. Perianal lesion was observed in approximately 18~20% patients with Crohn’s disease, and other common comorbidities included diabetes, cardiovascular and pulmonary illnesses. Notably, a certain proportion of patients with Crohn’s disease had previous smoking history or were currently smoking. In SSI group, there were 36.7% patients were previous or current smoker compared to 20% patients in non-SSI group (*p* = 0.03) (Table 1).

**Table 1:** Demographics and clinical features of patients in both groups.

	SSI group (n = 49)	Non-SSI group (n = 105)	*p*-value
Male (n, %)	33 (67.3%)	73 (69.5%)	ns
Age at definitive operation (yrs.)	41.3 ± 9.8	39.5 ± 11.5	ns
BMI (kg/m^2^)	19.6 ± 2.1	20.3 ± 2.9	ns
Duration of disease (yrs.)	4.4 ± 3.1	4.7 ± 3.5	ns
Past surgical history (times)	1.7 ± 0.8	1.9 ± 1.1	ns
None (n, %)	11 (22.4%)	26 (24.8%)	ns
Yes (n, %)	38 (77.6%)	79 (75.2%)	—
Comorbidity (n, %)	—	—	ns
Perianal lesion	10 (20.4%)	19 (18.1%)	—
Diabetes mellitus	4 (8.2%)	6 (5.7%)	—
Cardiovascular disease	3 (6.1%)	4 (3.8%)	—
Pulmonary disease	3 (6.1%)	4 (3.8%)	—
Renal disease	2 (4.1%)	2 (1.9%)	—
Others	0	1* (1.0%)	—
Tobacco usage (n, %)	—	—	0.03
Previous or current	18 (36.7%)	21 (20.0%)	—
Never	31 (63.3%)	84 (80.0%)	—

*The only one case of comorbidity was ankylosing spondylitis. Abbreviations: BMI, body mass index; ns, not significant.

We further compared lab test results of patients between two groups (Table 2). The mean CDAI (Crohn’s disease activity index) score were more than 150 when being admitted, indicating the existence of active disease status in patients of both groups. In contrast, the CDAI score declined to 70~80 before surgery due to effective medications that induced and maintained remission of disease. The serum level of WBC, platelet and albumin were similar between groups, while the mean Hb (hemoglobin) and pre-albumin levels in SSI group were lower than normal range, and was significantly lower than that in non-SSI group (*p* = 0.02), indicating a relatively high prevalence of anemia and malnutrition in patients of SSI group. Preoperative CRP (c-reactive protein) and ESR (erythrocyte sedimentation rate) were higher than normal range, implying inflammatory events in patients with Crohn’s disease. Preoperative procalcitonin were within normal range, suggesting non-infectious status or effective source control before surgery in these patients.

**Table 2:** Laboratory examinations of patients in both groups.

	SSI group (n = 49)	Non-SSI group (n = 105)	*p*-value
CDAI score on admission	179.3 ± 78.4	185.4 ± 105.1	ns
CDAI score before surgery	80.2 ± 61.1	75.3 ± 69.0	ns
Preoperative WBC (×10^9^/L)	8.3 ± 4.5	7.7 ± 5.6	ns
Preoperative Hb (g/L)	84.5 ± 13.6	99.5 ± 6.3	0.02
Preoperative platelet (×10^9^/L)	179.5 ± 70.4	165.3 ± 77.2	ns
Preoperative albumin (g/L)	34.1 ± 6.6	35.8 ± 8.1	ns
Preoperative pre-albumin (mg/L)	190.2 ± 78.9	265.5 ± 84.0	0.02
Preoperative CRP (mg/L)	23.5 ± 11.6	17.8 ± 9.0	ns
Preoperative ESR (mm/h)	18.4 ± 10.2	16.8 ± 14.9	ns
Preoperative procalcitonin (ng/ml)	0.3 ± 0.1	0.2 ± 0.1	ns

Abbreviations: CDAI, Crohn’s disease activity index; ns, not significant; WBC, white blood cell; Hb, hemoglobin; CRP, C-reactive protein; ESR, erythrocyte sedimentation rate.

Table 3 demonstrated medical management within 3 months prior to bowel resection. All enrolled patients received proper medications for the induction and maintenance of disease remission. Specifically, 5-ASA (5-amino salicylic acid) including sulfasalazine and mesalazine was the most commonly used drug for patients in both groups, followed by Tripterygium wilfordii polyglycoside (TWP, a traditional Chinese medication). Immunosuppressant such as AZA/6-MP (azathioprine/6-mercaptopurine) was applied in approximately 10% patients, while corticosteroids and anti-TNF agents were relatively less used in both groups. Notably, the majority of patients received preoperative enteral nutrition whereas more than half of patients received preoperative parenteral nutrition in our center. Approximately half of patients received preoperative antibiotics in both groups. No statistical difference of medications was found between groups.

**Table 3:** Medical management within 3 months prior to definitive bowel resection.

	SSI group (n = 49)	Non-SSI group (n = 105)	*p*-value
Medications* (n, %)	—	—	ns
5-ASA	31 (63.3%)	68 (64.8%)	—
Corticosteroid	4 (8.2%)	5 (4.8%)	—
AZA/6-MP	5 (10.2%)	12 (11.4%)	—
Tripterygium wilfordii polyglycoside	22 (44.9%)	30 (28.6%)	—
Anti-TNF antibody	3 (6.1%)	8 (7.6%)	—
Preoperative enteral nutrition (n, %)	44 (89.8%)	97 (92.4%)	ns
Preoperative parenteral nutrition (n, %)	32 (65.3%)	72 (68.6%)	ns
Preoperative antibiotics (n, %)	28 (57.1%)	51 (48.6%)	ns

*Since a patient may receive several drugs during this period, the total percentage was more than 100%. Abbreviations: ns, not significant; 5-ASA, 5-amino salicylic acid (mainly includes sulfasalazine and mesalazine); AZA/6-MP, azathioprine/6-mercaptopurine.

In SSI group, 32 (65.3%) patients received definitive surgery due penetration/fistula whereas the other 17 (34.7%) patients were due to stricture/obstruction. In contrast, 50 (47.6%) and 55 (52.4%) patients in non-SSI group received definitive surgery due to the above two reasons, respectively (*p* = 0.04). Approximately one third of definitive bowel resections were performed using laparoscopic technique in our center. Remarkably, the duration of operation was significantly shorter in non-SSI group compared to that in SSI group (*p* = 0.03). Intraoperative arterial blood gas analysis demonstrated significantly higher lactate level in SSI group (*p* = 0.02), but similar blood glucose level and transfusion rate between two groups. The number and type of anastomosis were similar between two groups as well (Table 4).

**Table 4:** Operative data of enrolled patients in both groups.

	SSI group (n = 49)	Non-SSI group (n = 105)	*p*-value
Reason for definitive operation (n, %)	—	—	0.04
Stricture/obstruction	17 (34.7%)	55 (52.4%)	—
Penetration/fistula	32 (65.3%)	50 (47.6%)	—
Operative approach (n, %)	—	—	ns
Laparoscopic-assisted	15 (30.6%)	36 (34.3%)	—
Laparotomy (including conversion to laparotomy)	34 (69.4%)	69 (65.7%)	—
Duration of operation (min.)	123.8 ± 30.7	92.6 ± 27.4	0.03
Intraoperative blood glucose (mmol/L)	8.3 ± 4.5	7.2 ± 3.6	ns
Intraoperative blood lactate (mmol/L)	2.0 ± 1.3	1.4 ± 0.9	0.02
Intraoperative blood transfusion (n, %)	3 (6.1%)	5 (4.8%)	ns
Number of anastomosis	1.2 ± 0.5	1.1 ± 0.5	ns
Type of anastomosis* (n, %)	—	—	ns
End-to-end	2 (3.4%)	2 (1.7%)	—
End-to-side	38 (64.4%)	80 (69.6%)	—
Side-to-side	19 (32.2%)	33 (28.7%)	—

*Since more than one anastomosis may be performed during an operation, the total number of anastomosis was more than the total number of patients. Abbreviations: ns, not significant.

All variables that has been found statistically different between groups were brought into subsequent logistic regression analysis. These variables included tobacco usage, preoperative Hb and pre-albumin level, reason for operation, duration of operation, and intraoperative lactate level. Among them, three elements were identified as risk factors for postoperative SSI, including preoperative pre-albumin level (95% CI: 0.2~0.7; OR = 0.5; *p* = 0.03), duration of operation (95% CI: 2.3~9.5; OR = 3.8; *p* = 0.03) and intraoperative lactate level (95% CI: 1.7~7.2; OR = 3.4; *p* = 0.04) (Table 5).

**Table 5:** Risk factors for postoperative SSI using logistic regression analysis.

	95% CI	OR value	*p*-value
Tobacco usage (previous or current)	—	—	ns
Preoperative Hb	—	—	ns
Preoperative pre-albumin	0.2 ~ 0.7	0.5	0.03
Reason for operation (penetration/fistula)	—	—	ns
Duration of operation	2.3 ~ 9.5	3.8	0.03
Intraoperative lactate	1.7 ~ 7.2	3.4	0.04

Abbreviations: ns, not significant.

## Discussion

Herein, we summarize the main findings of our study. By comparing 49 SSI cases and 105 non-SSI cases of CD patients receiving definitive bowel resection, we find a statistical difference of tobacco usage, preoperative Hb and pre-albumin level, reasons for operation, duration of operation and intraoperative lactate level between patients in two groups. Further logistical regression analysis identifies that preoperative pre-albumin, duration of operation and intraoperative lactate level are significant predictive factors of postoperative SSI in CD patients. These findings suggest us to correct preoperative hypoprealbuminemia, accelerate operation process and provide adequate intraoperative oxygenation for CD patients to reduce SSI risk after definitive bowel resection.

Due to transmural bowel inflammation, patients with Crohn’s disease usually undergo bowel resection because of bowel stricture or penetration. High recurrence and reoperation rates are major challenges in the management of Crohn’s disease. A recent study from Korean CD population reported cumulative reoperation rates of 31.8% and 57.2% within 10 and 15 years, respectively^10^. Our previous survey of Chinese CD patients reported accumulated reoperation rates of 38.0%, 52.2% and 58.7% after 1, 3 and 5 years, respectively^5^. More importantly, Riss *et al*. discovered that repeated intestinal resections independently increase the risk of postoperative recurrence of Crohn’s disease^11^, which further exacerbate the dilemma of performing surgical interventions for CD patients.

Surgical site infection is quite common in abdominal surgery, especially in gastrointestinal surgery including surgical removal of diseased bowel in Crohn’s disease^12^. SSI causes delayed postoperative recovery, aggravated incisional adhesion, and potential risk of incisional hernia. It is therefore extremely pivotal to prevent SSI after primary or second operations in CD patients, thereby providing better condition for future possible reoperations. However, very few studies focused on prognostic factors for postoperative SSI in CD. It is largely unknown how to reduce the risk of SSI pre- and intra-operatively and how to early predict the appearance of SSI in CD patients. Our study has clearly demonstrated that improve pre-albumin level before surgery, supply adequate oxygenation during surgery and expedite bowel resection would be beneficial to decrease the risk of SSI in CD patients.

In current study, all CD patients received preoperative management including induction and maintenance of remission by medications, enteral or parenteral nutritional support, physical rehabilitation and source control strategy. All these actions are helpful to reduce the disease activity. Remission of disease is required before definitive surgery. Our previous studies have demonstrated the efficacy of above strategies in inducing and maintaining remission of CD^4, 13–16^.

According to the newly released WHO recommendations, nutritional support is suggested to prevent SSI in patients who undergo major surgeries^6^. In our center, enteral nutrition combined with parental nutrition (if necessary) are prescribed for the majority of CD patients, in order to improve nutritional status as well as to maintain the remission of CD^13–15^. Current study further discovers the value of nutritional support in preventing SSI.

In consistent to our findings, the WHO panel recommends high fraction (80%) of inspired oxygen intraoperatively to prevent SSI^7^, our data identifies intraoperative lactate level as an independent predictive factor for SSI, and confirmed the importance of intraoperative oxygenation in reducing SSI in CD patients. Although discontinuation of immunosuppressive agents is not encouraged by WHO panel, the application of immunosuppressant in CD patients is quite limited in our center. In contrast, 5-ASA and TWP (a traditional Chinese medication of which the efficacy and mechanism in CD has been investigated previously^16–19^) are the most commonly used drugs for CD. Our previous investigation identified that induction of Foxp3( + ) Tregs could be therapeutic mechanism of TWP in CD^20^.

We are aware of our limitations. First, this is a single-center retrospective case-control study, leading to potential selection bias that can hardly be avoided. Second, several known risk factors of SSI (such as incisional protector and intraoperative hypothemia) were not fully evaluated in our study. Possible interacting effect might exist that affect the reliability of our analysis. Third, follow-up data was not fully available from all patients, and therefore we could not evaluate the effect of SSI in long-term outcome in CD patients. Nevertheless, our study has provided a comprehensive exploration towards the risk factors of postoperative SSI in CD patients receiving definitive bowel surgery. We expect future prospective studies with large sample size could provide more strategies in the prevention of SSI in patients with Crohn’s disease.
